# Supplementary material for: Universal, untargeted detection of bacteria in tissues using metabolomics workflows
Source: Nat Commun. 2025 Jan 2;16:165. doi: 10.1038/s41467-024-55457-7 (PMC11697447; doi:10.1038/s41467-024-55457-7)
Supplement: Supplementary file 1 — Supplementary Information [file 41467_2024_55457_MOESM1_ESM.pdf]

Supplementary Information

Universal, untargeted detection of bacteria in tissues using metabolomics workflows

Wei Chen[1], Min Qiu[1], Petra Paizs[2], Miriam Sadowski[3], Toma Ramonaite[2], Lieby Zborovsky[1], Raquel Mejias-Luque[4], Klaus-Peter Janßen[5], James Kinross[6], Robert D. Goldin[2], Monica Rebec[7], Manuel Liebeke[3,8], Zoltan Takats[2,9], James S. McKenzie[2,Δ,*], Nicole Strittmatter[1,Δ,*]

Affiliations:

[1] Department of Bioscience, School of Natural Sciences, Technical University of Munich, Garching, Germany

[2] Department of Metabolism, Digestion and Reproduction, Imperial College London, London, United Kingdom

[3] Department of Symbiosis, Max Planck Institute for Marine Microbiology, Bremen, Germany

[4] Institute for Medical Microbiology, Immunology and Hygiene, School of Medicine and Health, Technical University of Munich, Munich, Germany.

[5] Department of Surgery, School of Medicine and Health, Technical University of Munich, Munich, Germany

[6] Department of Surgery and Cancer, Imperial College London, London, United Kingdom

[7] North West London Pathology, Imperial College Healthcare NHS Trust, London, United Kingdom

[8] Department for Metabolomics, Institute for Human Nutrition and Food Science, University of Kiel, Kiel, Germany

[9] Department of Immunomedicine, University of Regensburg, Regensburg, Germany

[Δ]Authors contributed equally.

*Correspondence to Nicole Strittmatter, nicole.strittmatter@tum.de, T: +49 89 28913321 and James McKenzie, j.mckenzie@imperial.ac.uk.

Content:

TSMs grouped by phylogenetic level and respective group of bacteria, Overall database size versus number of TSMs, Zoomed MS images for a selection of the taxon-specific markers (TSMs) of sample A35 centre of the tumour, Single ion images of taxon-specific markers (TSMs) detected in sample A35 centre of tumour and A64 5cm, TSM markers detected in colorectal DESI MSI vs faecal LC-MS datasets and the corresponding Venn diagram, Hematoxylin and eosin (H&E) staining images for tissues in Figure 6, Comparison between MSI and FISH images of murine jejunum tissue, Supplementary Methods, Instrumental parameters, Boxplots and ROC curves on different taxonomic levels for three different taxon-specific markers (TSMs) from the Bacteroidetes phylum, Validation datasets.

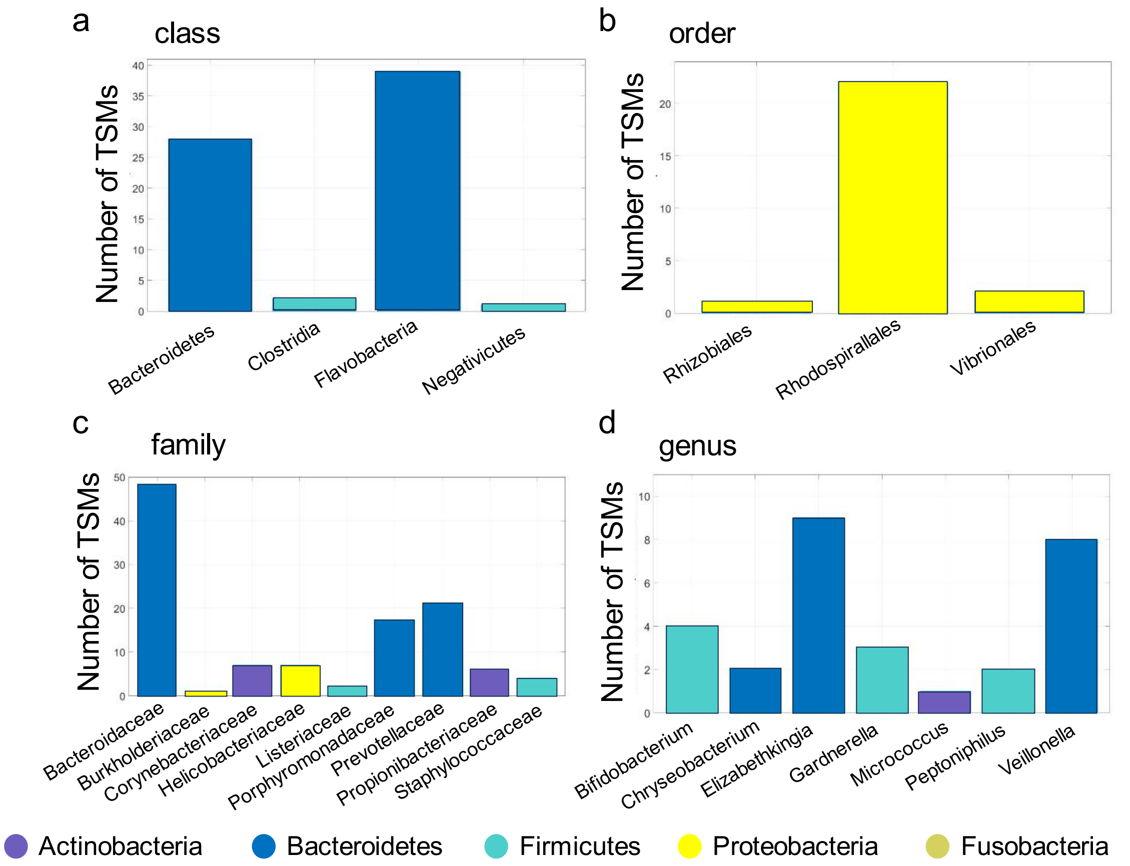

Supplementary Figure 1. TSMs grouped by phylogenetic level and respective group of bacteria. a class-level, **b** order, **c** family, and **d** genus. Bars coloured by phylum (purple: Actinobacteria, blue: Bacteroidetes, petrol: Firmicutes, yellow: Proteobacteria, green: Fusobacteria.

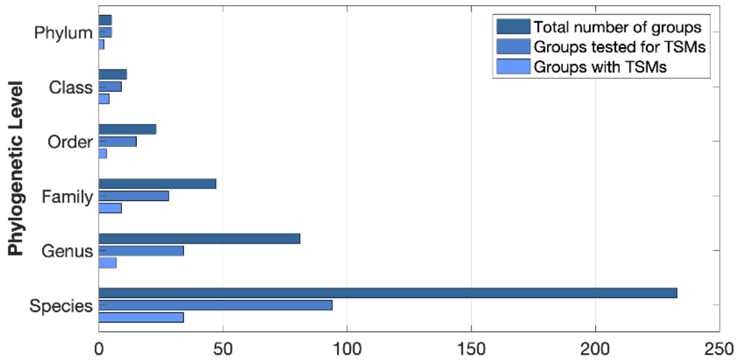

Supplementary Figure 2. Overall database size versus number of TSMs. Comparison of the total number of groups on each phylogenetic level (dark blue) versus those groups actually tested for TSMs due to the applied selection criteria (at least 3 files per group, at least two species for genus etc, mid blue) and the number of groups for which ultimately TSMs were found (light blue). Source data are provided as a Source Data file.

Of those species listed in Supplementary Data 1 (Training dataset), only those containing more than 3 database entries and at least two groups per level (e.g. two species on genus level) were tested for TSMs (each TSM was however tested against the whole database of 233 species). This results in 94 of 233 bacterial species tested for TSMs on the species level. Thus, TSMs could be found for 34 of the 94 (36%) eligible bacterial species. This is exemplified for each taxonomic level in Supplementary Fig. 2. The worst marker coverage was found on the phylogenetic level order and genus with 20% and 21%, respectively.

Feasibility of detecting TSM in colorectal cancer specimens using DESI-MSI

To demonstrate the applicability of these taxonomical markers for the detection of bacteria in complex biological, human-derived samples, we attempted to visualise the presence and distribution of bacteria in 44 human colorectal tissues recorded using DESI-MSI (see Supplementary Data 4 for access links). Tissue specimens were either from a tumour lesion or 5 to 10 cm distant. Bacteria cover the mucosal membranes in the gut and the effect of the gut microbial community on health and disease has been abundantly proven.[1,2] Sequencing-based data of the same tissues is available (Supplementary Data 5), however, tissues were halved to enable both analyses and thus the data is not strictly comparable.

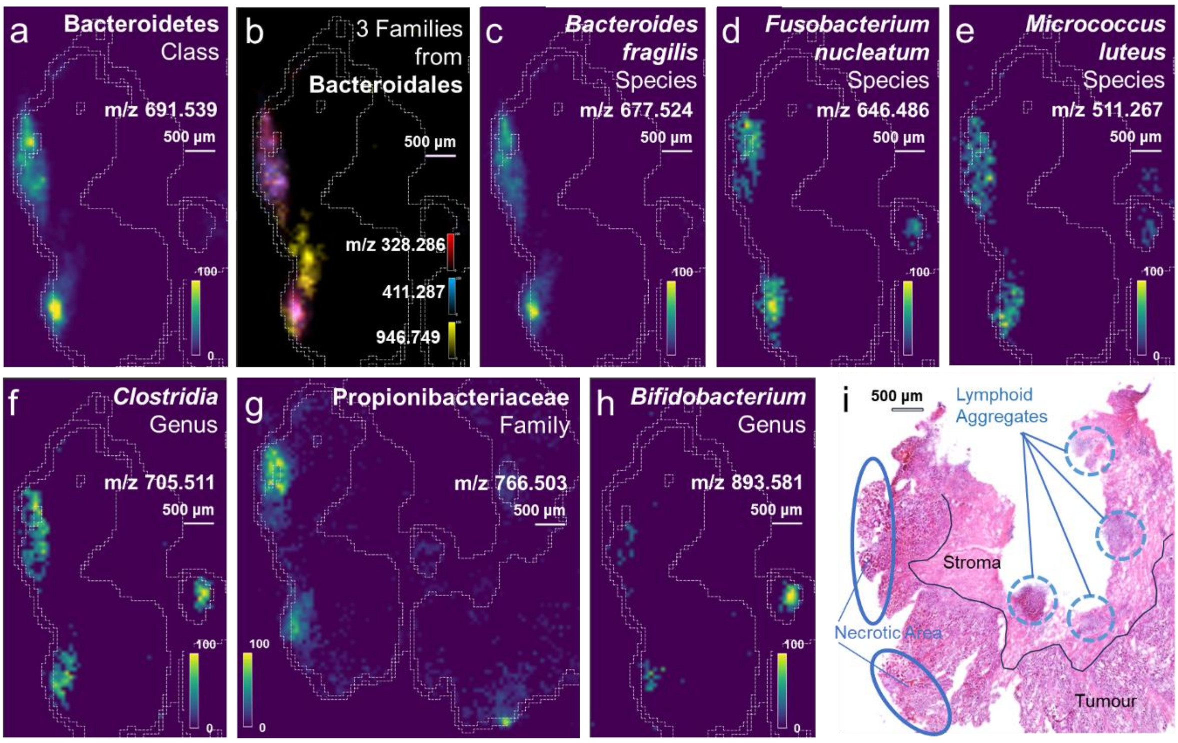

Supplementary Figure 3. Zoomed MS images for a selection of the taxon-specific markers (TSMs) of sample A35 centre of the tumour. a-**d**: TSMs for Gram-negative bacteria, **e**-**h**: for Gram-positive bacteria. Scale bars, 500 µm. **a** TSM for taxon Bacteroidetes, **b** Overlapping ion images of TSM for Bacteroidaceae, Prevontellaceae and Porphyromonadaceae, **c** TSM for Bacteroides fragilis, **d** TSM for *Fusobacterium nucleatum*, **e** TSM for *Micrococcus luteus*, **f** TSM for taxon Clostridia, **g** TSM for taxon Propionibacteriaceae, **h** TSM for taxon Bifidobacterium. Spatial resolution: 65µm. **i** Zoom into H&E staining of sample A35. Intensities of TSM were calculated with ±10 ppm mass tolerance and shown as relative intensities. For complete images see Supplementary Fig.4.

Among the cancerous specimens, bacteria were largely found localised in necrotic regions. However, bacteria were also frequently detected along healthy mucosa. Supplementary Fig. 3 shows the tissue type distribution of a cancerous tissue specimen A35 that originated from the centre of the tumour dissected during a right hemicolectomy. Histopathological examination revealed the presence of cancerous and stromal tissue, including smaller amounts of necrotic tissue and lymphoid aggregates. Mass spectra of the necrotic tissue

area as well as surrounding cancerous and stromal tissue are shown in Supplementary Fig. 3c and display a markedly different phospholipid composition for the necrotic area compared to viable tissues, namely a significantly reduced glycerophospholipid content and a variety of lower molecular weight sphingolipid-derived species in the mass range of *m/z* 500-700.

In total, 57 of 359 TSMs were detected with DESI-MSI in tumour tissue slide A35 CT. 49 TSMs were detected for Gram-negative bacteria and nine for Gram-positive. For Gram-negative bacteria, one marker was found for *Fusobacterium nucleatum* (belonging to phylum Fusobacteria, Supplementary Fig. 3d), one for order Rhodospirillales (phylum Proteobacteria), while all other markers were found for taxa under the phylum Bacteroidetes. Among these, two were found significant at discriminating at the phylum level for Bacteroidetes, seven TSMs were associated with the class Flavobacteria, and the remaining 38 were all within the class Bacteroidetes. Members of the Bacteroidetes phylum were reported to be accountable for up to 50% of the gut microbial community in metagenomic studies[1,3], and our own 16S rRNA sequencing analyses of colorectal cancer tissues echoed similar results of Bacteroidetes dominance, as shown in Supplementary Data 5**.**

Single-ion images for a subset of TSMs associated with Bacteroidetes are displayed in Supplementary Fig. 3a-c. The marker for the Bacteroidetes class at *m/z* 691.539 is distributed in two necrotic regions on the left side of the MS image. Focusing on the subclass of Bacteroidetes (Supplementary Fig. 3b), *m/z* 328.286 (identified as dihydroceramide) is specific for the Bacteroidaceae family and is showing co-localised distribution to the TSM for the Prevotellaceae family at m/z 411.287 (displayed in red and blue). *m/z* 946.749 shown here in yellow (iso-C15:0-substituted phosphoglycerol dihydroceramide) is a TSM specific for the family Porphyromonadaceae. The distribution of its homologue signal series with *m/z* 918.717, 932.735, 946.749, and 960.764 (Supplementary Fig. 3c) differs from those markers for Bacteroidaceae and Prevotellaceae, located predominantly above the second necrotic area, suggesting a succinct distribution of Porphyromonadaceae. The distribution of TSMs for Bacteroidaceae and Prevotellaceae in Supplementary Fig. 3b while largely similar shows a different hotspot pattern in the first necrotic region, which links to the two separated hotspots found in this region for Bacteroidetes (Supplementary Fig. 3a). This example demonstrates the spatial heterogeneity of subclasses under a higher taxonomic level. MS images of TSM for species *B. fragilis* from Bacteroidaceae are shown in Supplementary Fig. 3c**.** This ceramide phosphatidylethanolamine at *m/z* 677.524 was also found by Frankfater *et al.* in the *B. fragilis* group cell extracts.[4]. *B. fragilis* is known as one of the most common bacterial species in clinical specimens and is essential for healthy gastrointestinal tract functions[5,6] while enterotoxigenic *B. fragilis* is thought to play a key role in the occurrence and progression of colorectal cancer[7,8]. In Supplementary Fig. 3d, a third colonised hotspot is

found to the right of the central lymphoid aggregate for the marker of *Fusobacterium nucleatum*, which does not appear to be colonised by Bacteroidetes. While enterotoxigenic *B. fragilis* is thought to play a key role in the occurrence and progression of colorectal cancer[7,8].[7,8]. In Supplementary Fig. 3d, a third colonised hotspot is found to the right of the central lymphoid aggregate for the marker of *Fusobacterium nucleatum*, which does not appear to be colonised by Bacteroidetes.

Of the eight TSMs detected for Gram-positive bacteria, Firmicutes and Actinobacteria phyla supplied four markers each. Markers for Actinobacteria covered three different classes: Micrococcales, Actinomycetales, and Bifidobacteriales, as shown in Supplementary Fig. 3e**,** g, h. The Gram-positive bacteria are more diverse in their spatial distribution patterns. For the Clostridia class (part of phylum Firmicutes, Supplementary Fig. 3f) and Bifidobacterium genus (part of phylum Actinobacteria, Supplementary Fig. 3h)**,** both *m/z* 705.511 and 893.581 were found to have a third hotspot near the central lymphoid aggregate, whereas for *Micrococcus luteus* an expanded but attenuated distribution zone was detected. Propionibacteriaceae (Supplementary Fig. 3g) and Bifidobacterium (Supplementary Fig. 3h) are both members of the Actinobacteria phylum but exhibit unique spatial distribution patterns. In Supplementary Fig. 3g, *m/z* 766.503 shows the most extensive distribution pattern suggesting colonisation with strong growth of Propionibacteriaceae over wider tissue areas. A small and strong hotspot of this marker can be seen at the bottom right periphery which did not seem to be colonised by detectable amounts of Bacteroidetes and other phyla. Bifidobacterium seemed to mainly colonise in the vicinity of the central lymphoid aggregate. All of these detected bacterial classes are capable of living under anaerobic conditions and were reported to be major components of the human gut microbiome.[1] An example of healthy colorectal tissue specimens is shown in Supplementary Fig. 5.

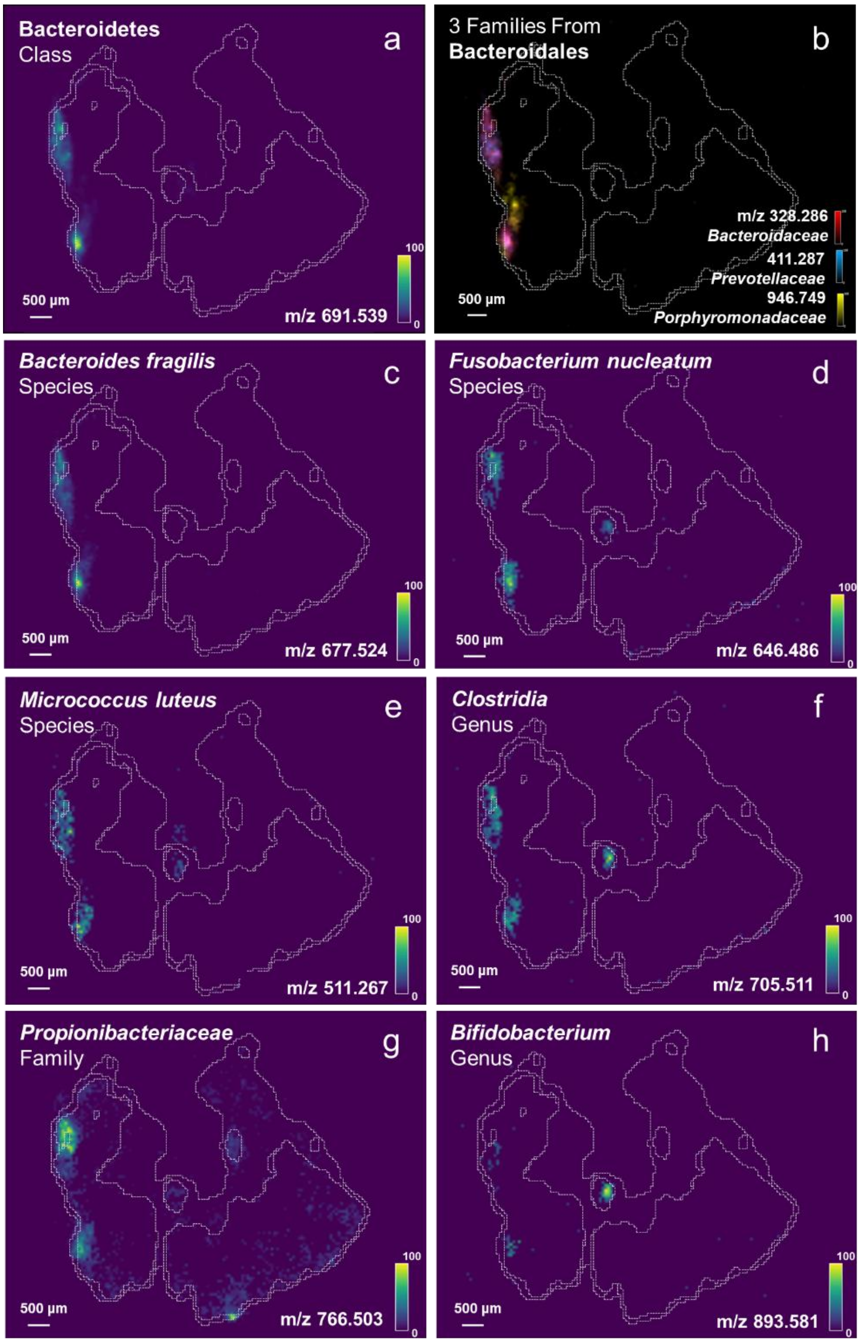

Supplementary Figure 4. Single ion images of taxon-specific markers (TSMs) detected in sample A35 centre of tumour. a-d TSMs for Gram-negative bacteria, **e-h** for Gram-positive bacteria. Scale bars, 500 µm. **a** TSM for taxon Bacteroidetes, **b** Overlapping ion images of TSM for Bacteroidaceae, Prevontellaceae and Porphyromonadaceae, **c** TSM for *Bacteroides fragilis*, **d** TSM for *Fusobacterium nucleatum*, **e** TSM for *Micrococcus luteus*, **f** TSM for taxon Clostridia, **g** TSM for taxon Propionibacteriaceae, **h** TSM for taxon Bifidobacterium. Spatial resolution: 65 µm.

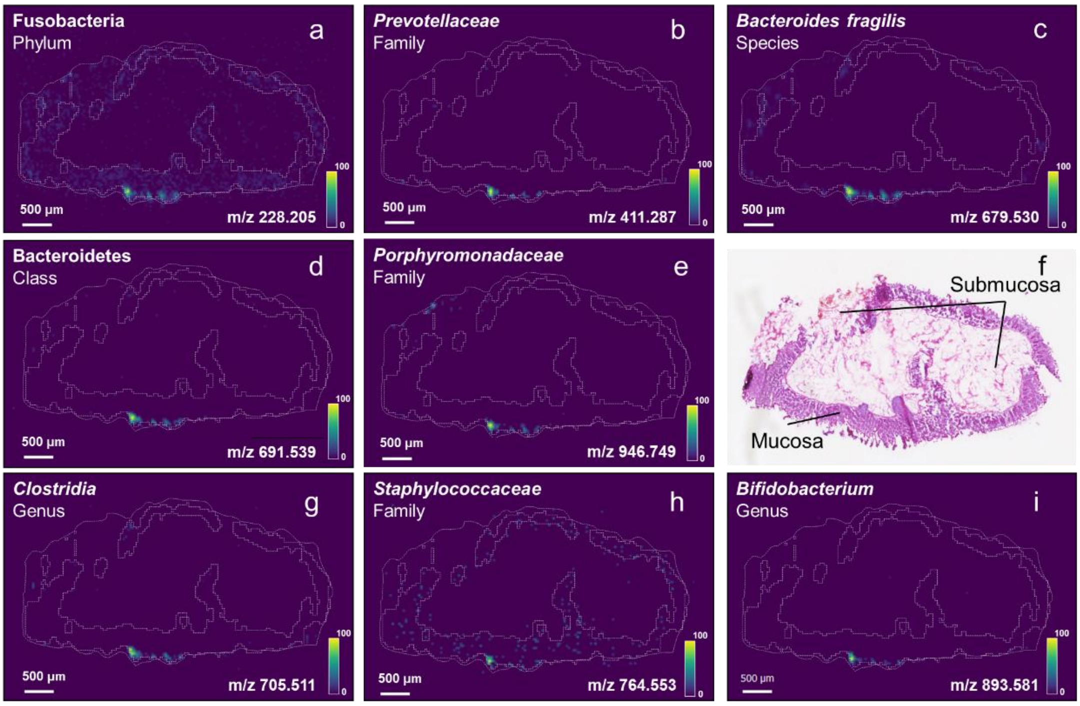

Supplementary Figure 5. Single ion images of taxon-specific markers (TSMs) detected in healthy colorectal tissue specimen A64 5cm and H&E image of the same tissue section. **a**-**e** for Gram-negative bacteria, **g**-**i** for Gram-positive bacteria. Scale bars, 500 µm. **a** TSM detected for taxon Fusobacteria, **b** TSM detected for Prevotellaceae, **c** TSM detected for *Bacteroides fragilis*, **d** TSM detected for taxon Bacteroidetes, **e** TSM detected for taxon Porphyromonadaceae, **g** TSM detected for taxon Clostridia, **h** TSM detected for taxon Staphylococcaceae, **i** TSM detected for Bifidobacterium. Spatial resolution: 85 µm. **f** H&E image of the same tissue section.

Supplementary Fig. 5 displays the tissue type distribution of a healthy tissue specimen from a right hemicolectomy, 5 cm distant from the centre of the tumour. Histopathological examination revealed healthy mucosa and submucosa, separated by the muscularis mucosae layer. TSMs detected in this sample are similar to those previously detected in the cancerous lesion, comprising mainly gastrointestinal tract bacteria of the Bacteroidetes phylum. In this specimen, TSMs were predominantly localised in several hotspots at the bottom mucosa periphery. Generally, less numerous and less intense TSM signals were observed in this healthy specimen than in the case of the previous tumour example. This is tentatively attributed to the healthy immune response that restricts unlimited bacterial growth as is more likely to happen in necrotic tissue areas. The highest number of TSMs could again be detected for the Bacteroidetes phylum (45/67, Supplementary Fig. 5b-e). Markers for bacteria from Fusobacteria (Supplementary Fig. 5a) as an example), Actinobacteria (Supplementary Fig. 5i), Firmicutes (Supplementary Fig. 5g, h) and Proteobacteria phylum were additionally detected.

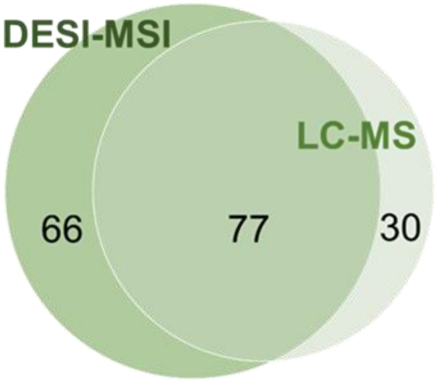

Supplementary Figure 6. Venn diagram showing overlap of TSMs detected in imaging-based and LC-MS-based analysis in colorectal tissue and faeces, respectively. For DESI-MSI, n=44 samples; for LC-MS, n=48 samples. More details can be found in Supplementary Data 6.

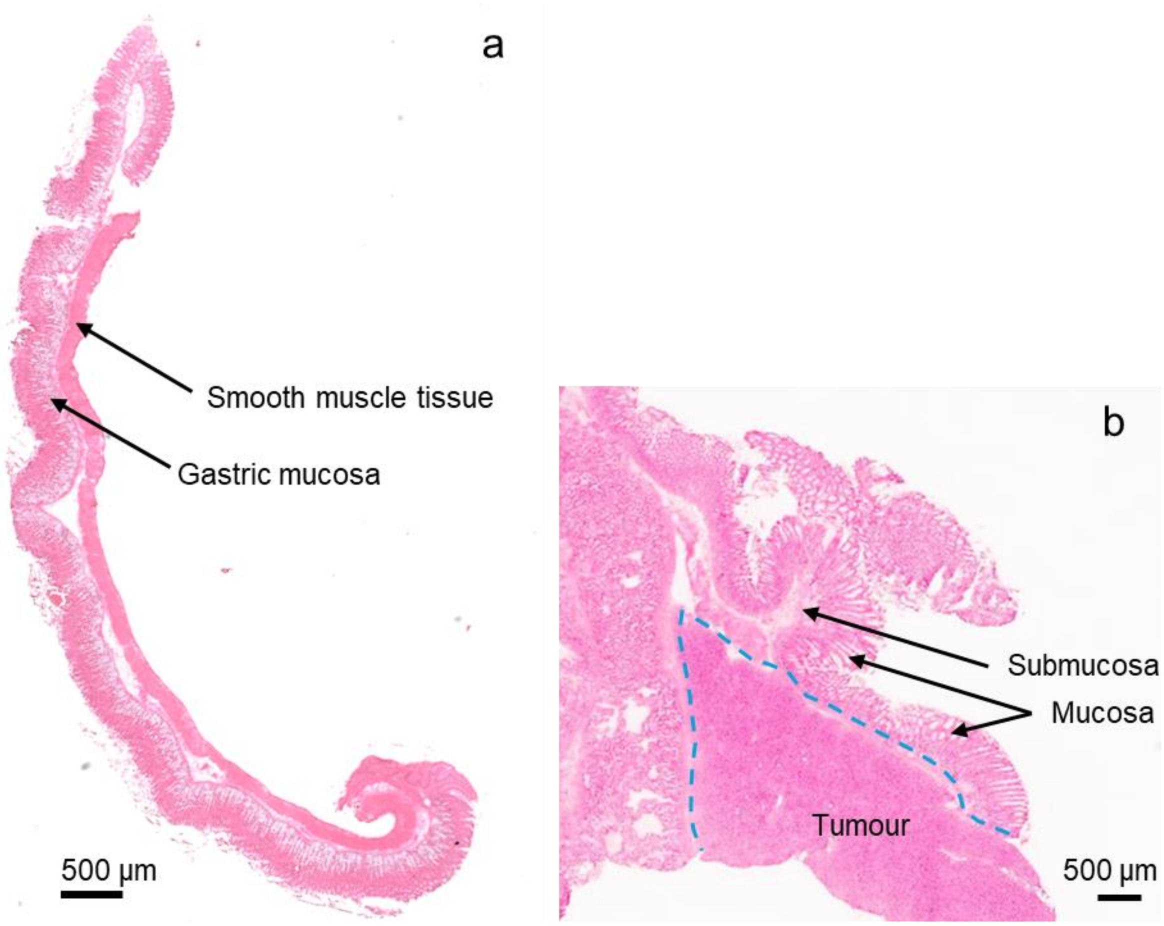

Supplementary Figure 7. Hematoxylin and eosin (H&E) staining images for tissues in Figure 6. a Gastric tissue of a C57BL/6 mouse (female, 8 weeks old), 1-week post-infection with *H.pylori* (PMSS1). The neighbouring tissue section of the section in Figure 6a was used. Scale bars, 500 µm. **b** Tumour tissue of a mouse model for colorectal cancer (Apc$^{1638N/wt}$ x pvillin-Kras^{V12G}). The same tissue section from Figure 6e was used. Scale bars, 500 µm.

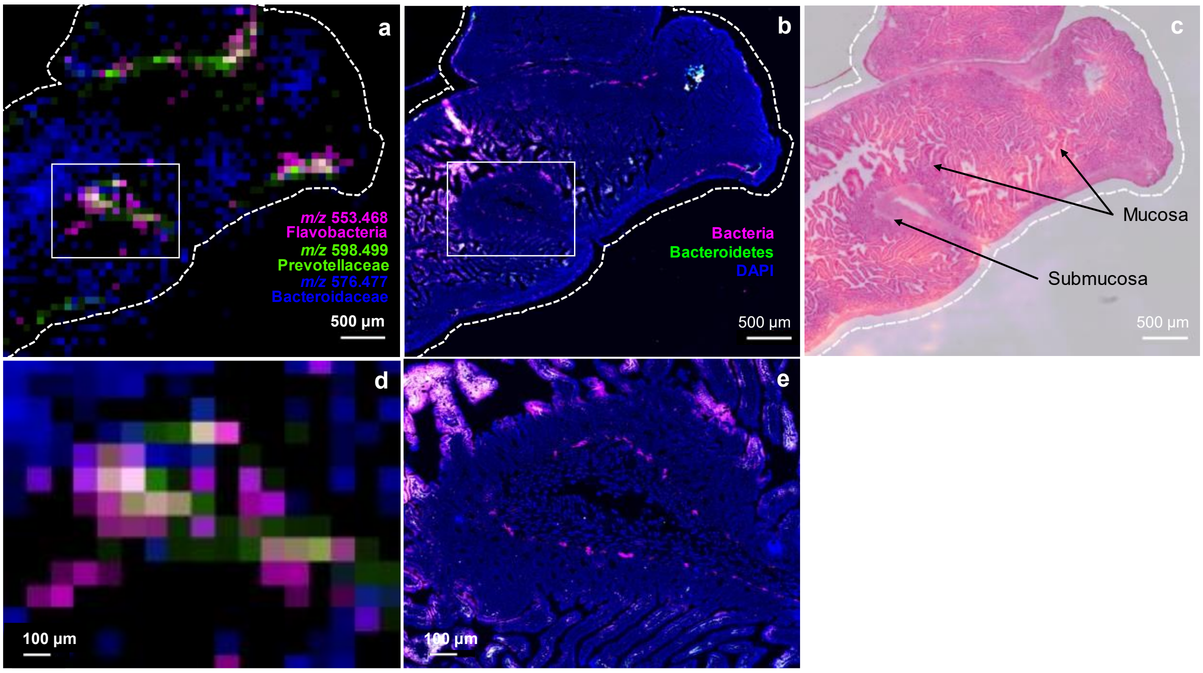

Supplementary Figure 8. Comparison between MSI and 16S rRNA FISH images of murine jejunum tissue. **a** Overlayed DESI-MS image for different TSMs. MS spatial resolution: 80 µm. Magenta: *m/z* 553.468 (Flavobacteria, Class), blue: *m/z* 576.477 (Bacteroidaceae, Family), green: 598.499 (Prevotellaceae, Family). Scale bars, 500 µm. **b** 16S rRNA FISH image of neighbouring tissue section to **a**. Scale bars, 500 µm. Blue: DAPI, magenta: Eub338 bacteria FISH probe, green: CF319a FISH probe for phylum Bacteroidetes. Mass tolerance for generation of the MS images: 10 ppm. One slide for two murine jejunum tissue was analysed and the results were similar, showing good correspondence between the DESI-MS images and the FISH images. **d** Zoomed overlayed DESI-MS image, the zoomed area is boxed in **a.** Scale bars, 100 µm. **e** Zoomed FISH image, the zoomed area is boxed in **b**. Scale bars, 500 µm. **c** Image from hematoxylin and eosin (H&E) staining for tissue section in **a**, obtained after DESI-MSI analysis, overlapped with total ion current (TIC) DESI-MS image. Scale bars, 500 µm. For 16S rRNA FISH images, the intensity threshold of individual colour channels was adjusted correspondingly to merge three colour channels and avoid overexposure. Overlapped DESI MSI images were generated with a relative intensity scale bar for individual *m/z* values without adjustment.

Supplementary Methods:

DESI-MSI of human colorectal tissue specimens. DESI-MS imaging of human colorectal tissue specimen was performed using a home-built DESI source mounted to a Thermo Scientific Exactive instrument (Thermo Fisher Scientific Inc., Bremen, Germany) operated in negative ion mode (full scan, *m/z* 200-1000) at a mass resolution of 100,000 (Instrument parameters are given in Supplementary Table 1). MS instrument was calibrated using the commercially available Thermo Fisher Scientific PierceTM calibration mix before use with a home-built nanoelectrospray ionisation source. Tissues were resected during a single-centre study with full ethical approval by the institutional review board at Imperial College Healthcare NHS Trust. This study was granted full ethical approval by the institutional review board at Imperial College Healthcare NHS Trust (REC reference number 07/H0712/112). Patients underwent hemicolectomy and tissue specimens were taken from the centre of the tumour (CT), 5 cm and 10 cm distant from the tumour (regarded as healthy); details on surgical procedure and tissue site for each sample can be found in Supplementary Data 4. Fresh frozen tissues were cryosectioned with a thickness of 10-20 µm and thaw mounted onto Superfrost glass slides and stored under -80 °C until analysis. In short, methanol-water (95:5 v/v) was used as electrospray solvent at a flow rate of 1.5 µL/min and spray voltage of -4.5 kV. Nitrogen N4.8 was used as nebulising gas at a pressure of 7 bars. The distance between the sprayer and the sample surface was 1.5 mm, while the distance between the sprayer tip and the MS inlet was 14 mm. All samples are deposited and publicly accessible on the Metaspace and MetaboLights platforms. Data have been deposited in the MetaboLights database under accession code MTBLS289. Access links are supplied in Supplementary Data 4.

Pre-processing of imaging data. The 359 specific *m/z* values were used to extract ion images from 44 DESI mass spectrometry imaging files of human colorectal tissue. Raw spectral data were converted to .imzML format in profile mode.[9] To extract ion images from each MSI, recalibration of each spectrum was first performed by linearly interpolating a ppm shift between known reference peaks at theoretical *m/z* 255.2330 (FA 16:0) and 885.5499 (PI 38:4), peaks which are almost universally present in colorectal tissue. From the recalibrated spectrum, the intensity of peaks within ±10 ppm of the 359 specific *m/z* values were recorded.

Spatial analysis. Spatial tests were applied to all ion images to identify features with noise-like distributions. The following tests were applied: a) mean tissue intensity at least three times greater than mean background intensity; b) a minimum of three non-zero diagonally connected pixels; c) a non-random spatial distribution in images with 50 or fewer non-zero pixels. For an image with n non-zero pixels, 2500 binary images with n randomly distributed non-zero pixels were generated. The summed pairwise city block distances for each binary image were determined. Binarised ion images with a cumulative city block distance less than the median of these 2500 distances were considered non-random and passed the test.

Liquid chromatography MS of faecal samples. Faecal samples were collected in a prospective observational pilot study performed at Imperial College London NHS Trust, UK. Patients undergoing elective surgical resection for primary Right-sided colorectal cancers (RCRCs) were recruited, and all were treatment naïve. The sample collection period is from February 2020 to May 2021. Patients undergoing resectional surgery have consented under Imperial College London tissue bank (SUR_JK_17_046 PI: Mr. James Kinross). Of the total of 48 Intra-luminal faecal samples, 20 were collected from females and 28 from males.

Faecal samples underwent a monophasic isopropanol (IPA) extraction slightly modified from Sarafian, et al [10]. Pre-cooled IPA in a 1:5 (v/v) sample/solvent ratio was added to each

Eppendorf of the pooled faecal samples. Faecal samples in IPA were vortexed for 1 minute and incubated for 2 hours at 4˚C. In the next step, five steel beads were added for bead beating (BertinTechnologies) at 6500 G, 2x45 seconds. The samples were then vortexed for one minute and placed at 4˚C for protein precipitation overnight. The samples were then centrifuged (Eppendorf, Centrifuge5417R, Germany) at 14,000 G for 10 minutes at 4˚C. The supernatant was transferred into a clean 2 ml Eppendorf vial, and the centrifugation step was repeated at 14,000 G for 10 minutes at 4˚C. The supernatant was transferred into a Waters Polypropylene Screw Neck Vial, 12 x 32 mm, 300 µL HPLC vial (Waters Corporation, UK) for UHPLC-MS analysis and stored at - 80 ˚C. Furthermore, a quality control (QC) and blank samples were prepared to monitor the performance of the instrument throughout the analysis. Samples were acquired by UHPLC-MS using previously published analytical and quality control procedures to generate high-quality datasets.[11-13].

Assays comprised a reversed-phase chromatography tailored for the separation of lipophilic analytes (e.g., complex and neutral lipids) in both positive and negative ion modes. Following extraction, the samples were defrosted for 2 hours at 4˚C and centrifuged at 3896g for 10 minutes, 4˚C. An 80 µL aliquot of each sample was taken to create a pooled QC sample (study reference, SR). All samples were diluted 1:1 by adding a H_2O: IPA 1:4 containing standards (LPC(9:0/0:0); PC(11:0/11:0); FA(17:0); PE(15:0/15:0); PA(17:0/17:0); PG(15:0/15:0); PS(17:0/17:0); Cer(d18:1/17:0); DG(19:0/0:0/19:0); PC(23:0/23:0); TG(15:0/15:0/15:0); TG(17:0/17:0/17:0)), vortexed 10 seconds, shaken for 5 minutes at 1400rpm at 4˚C. Samples were then centrifuged for 10 minutes at 3896 g at 4˚C and split into two vials, one for each polarity. An additional set of SR sample dilutions was created (10x 100%, 5x 80%, 3x 60%, 3x 40%, 5x 20%, 10x 1%). The SR sample was injected at regular intervals throughout the analytical run to ensure system stability and for subsequent pre-processing. The SR sample dilutions were analysed at the start and end of the sample analyses for assessment of analyte response. Samples were acquired in an order randomised against key study design elements. 2 µL of the sample were injected for negative polarity and 1 µL for positive polarity onto a Waters Acquity UPLC BEH C8, 1.7 µm, 2.1 × 100 mm column (Waters Corp., Milford, MA, USA) at 55 °C using an ACQUITY UPLC system (Waters Corp., Milford, MA, USA). The mobile phase's composition was as follows: A) water:isopropanol: acetonitrile (2:1:1), 5 mM ammonium acetate, 0.05% acetic acid, 20 µM phosphoric acid; B) isopropanol: acetonitrile 1:1 , 5 mM ammonium acetate, 0.05% acetic acid. Each sample was resolved for 13.15 min at a flow rate of 0.6 mL/min. Starting conditions were 1% B, the gradient increased to 30% B over the first 2 min, followed by an increase to 90% B from 2 to 11.50 min, increased to 99.9% B from 11.50 to 12 min while increasing the flow rate to 1 mL/min, staying at 99.9% B for 0.5 min, the solvent composition then returned to starting conditions over 0.25 min until 15 min. The UPLC was linked to a Xevo G2-S oaTOF MS (Waters Corp., Manchester, UK) coupled via a Zspray electrospray ionization (ESI) source. The capillary voltage was set to 2 kV for positive polarity and 1.5 kV for negative polarity, and the sampling cone voltage to 25 V. The desolvation temperature was set to 600 °C 1000 L/h, the desolvation gas flow to 1000 L/h, source temperature to 120 °C and cone gas flow of 150 L/h. Accurate mass was maintained by acquisition of LockSpray interface of Leucine Enkephalin (*m/z* 236.1035 and 554.2615 in ESI−; *m/z* 278.1141 and 556.2771 in ESI+) at a concentration of 600 pg/µL in 1:1 H2O: Acetonitrile, scan time of 0.15 s over 4 scans, and an interval of 60 s. Data were collected from 50 to 2000 *m/z* in centroid mode. Data are deposited under MetaboLights identifiers MTBLS11775.

LC-MS data analysis. Raw data was converted to the mzML open-source format and signals below an absolute intensity threshold of 100 counts were removed using the MSConvert tool in ProteoWizard[15]. For profiling datasets, feature extraction was performed using XCMS[16] In addition, extraction of target metabolites from the raw profiling data was

performed using peakPantheR, an R package for targeted integration of chemical signals from LC-MS datasets.[17] Using peakPantheR, for each UPLC-MS dataset, empirical retention time and theoretical *m/z* values from an in-house database of metabolite/lipid annotations were integrated yielding complementary datasets of known and pre-assigned metabolites.[13]

DESI-MS for comparison of mass spectra obtained by REIMS and DESI-MS. 1.5 µL freshly cultured *B. fragilis* biomass was pipetted on Superfrost glass slides and stored under -80 °C until analysis after air drying. Measurement set-up can be found in the paragraph of DESI-MSI of human colorectal tissue specimens, instrument parameters are given in Supplementary Table 1.

Murine tissue samples for validation of markers in complex samples. The murine colorectal tumour tissue (compound transgenic strain $Apc^{1638N/wt}$ x $pvillin\text{-}Kras^{V12G}$ on a C57/BL6N background[14]) and murine jejunum tissue (BALB/c mouse) were offered by the Department of Surgery, School of Medicine and Health, Klinikum rechts der Isar, Technical University of Munich, Munich, Germany. Animal breeding, maintenance and experiments were carried out following institutional and national guidelines and regulations, following the 3R principles of humane animal experiments (licence 55.2-1-54-2532-158-2015). The animal model and tissue preparation process can be found in reference[14,15]. *Helicobacter pylori*-infected stomach tissue was received from the Institute of Medical Microbiology, Immunology and Hygiene, Technische Universität München, Munich, Germany. The experiments were approved by the Bavarian Government (Regierung von Oberbayern, ROB-55.2-2532.Vet_02-19-95) The female C57/BL6 mouse was purchased from Envigo and housed under specific pathogen-free conditions (SPF) with food and H_2O ad libitum. The mouse was infected twice with *Helicobacter pylori* (PMSS1) at a time interval of 2 days at the age of 6 weeks. It was given $2x10^8$ bacteria per infection in 200 µL BHI medium supplemented with 20% FCS by oral gavage. The mouse was sacrificed by cervical dislocation 1 week after infection. The oesophagus, intestine and forestomach were separated from the stomach and the stomach was opened along the greater curvature. The stomach was cut into several longitudinal sections, and the tissue was washed with H_2O. Thereafter, tissue sections were snap-frozen in liquid nitrogen.

DESI-MSI of murine tissues. Fresh frozen tissues were embedded in hydroxypropyl methylcellulose (HPMC) and polyvinylpyrrolidone (PVP) mix (3:1, m/m) as described in the reference[16] and cryosectioned with a thickness of 10 µm and thaw mounted onto Superfrost glass slides and stored under -80 °C until analysis. The MSI analysis was performed on Q Exactive Orbitrap Mass Spectrometers (Thermo Fisher Scientific Inc., Bremen, Germany). The mass resolution used for all measurements was set to 70,000 at 200 *m/z*. The spatial resolution of the imaging experiments was 75 µm, except for the murine jejunum tissue at 80 µm. The height distance between the DESI sprayer and the sample surface was set to 2 mm; the distance between the sprayer and the inlet capillary was 7 mm. All spectra were recorded in negative ion mode. Mass spectra were recorded in the mass range of *m/z* = 200–1,000 for the colorectal tumour tissue and jejunum tissue (*n*=1), and for the nine *Helicobacter pylori*-infected stomach tissue (*n*=2): *m/z* = 100–600. Methanol-water (95:5 v/v) was used as electrospray solvent at a flow rate of 1.3 µL/min and spray voltage of -4.5 kV with a spray angle of 80°. Nitrogen N4.8 was used as nebulising gas at a pressure of 7 bars. The inlet capillary temperature was 320 °C. S-lens RF level at 100. All Thermo raw mass spectrometric data were converted to mzML format first via MSConvertGUI[17] and to imzML format via imzML Converter [9] with centroid mode. MS images were generated using LipostarMSI 2.0.1. Overlapped DESI MSI images were generated with a relative intensity scale bar for individual *m/z* values without adjustment. The colour scale in the images consistently starts from black (indicating zero intensity) and linearly transitions to the

corresponding colour annotated in the figures for different channels. All samples are deposited and publicly accessible on the Metaspace and MetaboLights platforms. Access links are supplied in Supplementary Data 4.

Supplementary Table 1. Instrumental parameters used for REIMS and DESI analysis.

Parameter	Setting REIMS	Setting DESI
Injection time	1000 ms	1000 ms
Microscans	1	1
Ion mode	negative	negative
Mass range	150-2000	200-1000
Tube Lens Voltage	-160 V	-150 V
Capillary Voltage	-50 V	-50 V
Skimmer Voltage	-24 V	-24 V
Capillary Temperature	250 ºC	250 ºC
Automatic Gain Control	On	On
AGC Target	High dynamic range	High dynamic range
Resolution	50,000 at *m/z* 200	100,000 at *m/z* 200

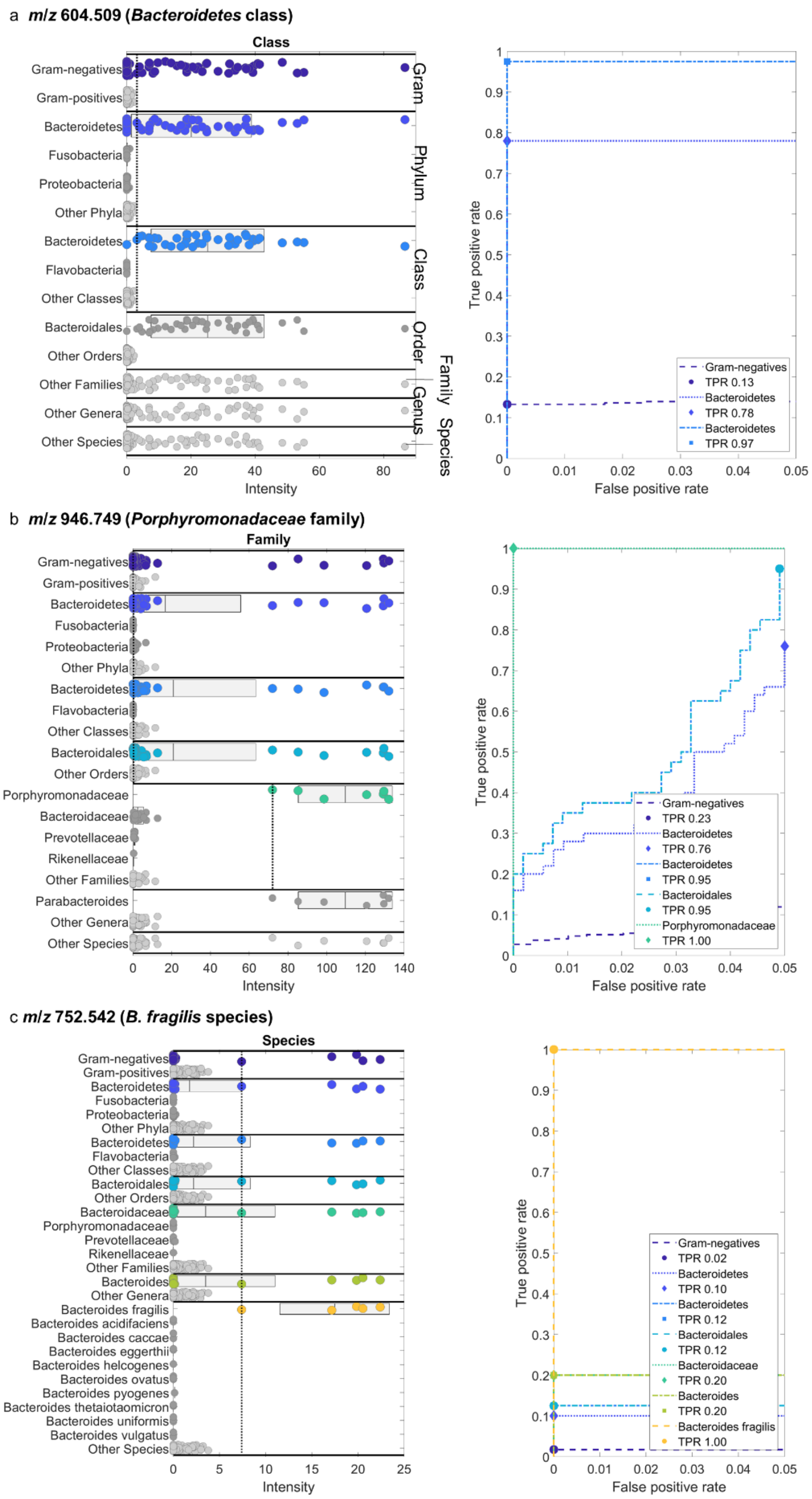

Supplementary Figure 9. Boxplots and ROC curves on different taxonomic levels for three different taxon-specific markers (TSMs) from the *Bacteroidetes* phylum. a *m/z* 604.509 (Bacteroidetes class, n=40 samples, *p*-value = 5.2004×10^{-129}), **b** *m/z* 946.749 (Porphyromonadaceae family, n=7 samples, *p*-value < 2.2251×10^{-308}), **c** *m/z* 752.542 (*Bacteroides fragilis* species, n=5 samples, *p*-value = 2.9106×10^{-112}). Box limits show the 25th and 75th percentiles, with the median marked in black. The intensities of TSMs in the boxplots were normalised to the total intensity over the 200-1000 m/z range. Source data are provided as a Source Data file.

Supplementary Table 2. Validation datasets. Access links to the METASPACE and MetaboLights datasets can be found in Supplementary Data 4.

**Ionisation**	**Analyser**	**Site**	**Description**	**Access via**
REIMS	Exactive classic	Imperial College, UK	3274 bacterial profiles from pure culture	MetaboLights MTLBS10328.
DESI-MSI	Exactive classic	Imperial College, UK	44 specimens of human colorectal cancer and healthy matched tissues (collected 2011-2013)	MetaboLights MTBLS289, METASPACE
REIMS	Waters Xevo G2-S oaTOF MS	Imperial College, UK	564 bacterial profiles spanning 38 species isolates	MetaboLights MTBLS11776
LC-MS	Waters Xevo G2-S oaTOF MS	National Phenome Center, ICL, UK	48 fecal samples from CRC patients (collected 2020-2021)	MetaboLights MTBLS11775
DESI-MSI	Q-Exactive	Technical University of Munich, Germany	*Helicobacter pylori*-infected murine stomach	MetaboLights MTBLS10846, METASPACE
DESI-MSI	Q-Exactive	Technical University of Munich, Germany	Murine jejunum and colon (incl. cancer)	MetaboLights MTBLS10846, METASPACE

Reference

1 Eckburg, P. B. *et al.* Diversity of the Human Intestinal Microbial Flora. *Science* **308**, 1635-1638 (2005). https://doi.org:doi:10.1126/science.1110591

2 Leviatan, S., Shoer, S., Rothschild, D., Gorodetski, M. & Segal, E. An expanded reference map of the human gut microbiome reveals hundreds of previously unknown species. *Nature Communications* **13**, 3863 (2022). https://doi.org:10.1038/s41467-022-31502-1

3 Turnbaugh, P. J. *et al.* A core gut microbiome in obese and lean twins. *Nature* **457**, 480-484 (2009). https://doi.org:10.1038/nature07540

4 Frankfater, C. F., Sartorio, M. G., Valguarnera, E., Feldman, M. F. & Hsu, F.-F. Lipidome of the Bacteroides Genus Containing New Peptidolipid and Sphingolipid Families Revealed by Multiple-Stage Mass Spectrometry. *Biochemistry* **62**, 1160-1180 (2023). https://doi.org:10.1021/acs.biochem.2c00664

5 Zhang, Z., Tang, H., Chen, P., Xie, H. & Tao, Y. Demystifying the manipulation of host immunity, metabolism, and extraintestinal tumors by the gut microbiome. *Signal Transduction and Targeted Therapy* **4** (2019). https://doi.org:10.1038/s41392-019-0074-5

6 Chan, J. L. *et al.* Non-toxigenic Bacteroides fragilis (NTBF) administration reduces bacteria-driven chronic colitis and tumor development independent of polysaccharide A. *Mucosal Immunology* **12**, 164-177 (2019). https://doi.org:10.1038/s41385-018-0085-5

7 Haghi, F., Goli, E., Mirzaei, B. & Zeighami, H. The association between fecal enterotoxigenic B. fragilis with colorectal cancer. *BMC Cancer* **19**, 879 (2019). https://doi.org:10.1186/s12885-019-6115-1

8 Dahmus, J. D., Kotler, D. L., Kastenberg, D. M. & Kistler, C. A. The gut microbiome and colorectal cancer: a review of bacterial pathogenesis. *Journal of Gastrointestinal Oncology* **9**, 769-777 (2018).

9 Race, A. M., Styles, I. B. & Bunch, J. Inclusive sharing of mass spectrometry imaging data requires a converter for all. *J Proteomics* **75**, 5111-5112 (2012). https://doi.org:10.1016/j.jprot.2012.05.035

10 Sarafian, M. H. *et al.* Objective set of criteria for optimization of sample preparation procedures for ultra-high throughput untargeted blood plasma lipid profiling by ultra performance liquid chromatography–mass spectrometry. *Analytical chemistry* **86**, 5766-5774 (2014).

11 Izzi-Engbeaya, C. *et al.* The effects of kisspeptin on β-cell function, serum metabolites and appetite in humans. *Diabetes, Obesity and Metabolism* **20**, 2800-2810 (2018).

12 Lewis, M. *et al.* An open platform for large scale LC-MS-based metabolomics. (2022).

13 Lewis, M. R. *et al.* Development and application of ultra-performance liquid chromatography-TOF MS for precision large scale urinary metabolic phenotyping. *Analytical chemistry* **88**, 9004-9013 (2016).

14 Janssen, K. P. *et al.* APC and Oncogenic KRAS Are Synergistic in Enhancing Wnt Signaling in Intestinal Tumor Formation and Progression. *Gastroenterology* **131**, 1096-1109 (2006). https://doi.org:https://doi.org/10.1053/j.gastro.2006.08.011

15 Buck, A. *et al.* Distribution and quantification of irinotecan and its active metabolite SN-38 in colon cancer murine model systems using MALDI MSI. *Analytical and Bioanalytical Chemistry* **407**, 2107-2116 (2015). https://doi.org:10.1007/s00216-014-8237-2

16 Dannhorn, A. *et al.* Universal Sample Preparation Unlocking Multimodal Molecular Tissue Imaging. *Analytical Chemistry* **92**, 11080-11088 (2020). https://doi.org:10.1021/acs.analchem.0c00826

17 Holman, J. D., Tabb, D. L. & Mallick, P. Employing ProteoWizard to Convert Raw Mass Spectrometry Data. *Curr Protoc Bioinformatics* **46**, 13.24.11-13.24.19 (2014). https://doi.org:10.1002/0471250953.bi1324s46
