# Supplementary material for: Universal, untargeted detection of bacteria in tissues using metabolomics workflows
Source: Nat Commun. 2025 Jan 2;16:165. doi: 10.1038/s41467-024-55457-7 (PMC11697447; doi:10.1038/s41467-024-55457-7)
Supplement: Supplementary file 3 — Description of Additional Supplementary Files [file 41467_2024_55457_MOESM3_ESM.pdf]

Description of Additional Supplementary Files

Supplementary Data 1. Composition of the training and internal validation dataset. The file names and then taxonomy from Gram to species are given. In the final column, the dataset is split into the 597 observations used for training and the remaining 2677 files used for validation.

Supplementary Data 2. Full list of all 359 TSMs. Phylogenetic levels, p-values, and AUC values are included. AUC: Area Under Curve, calculated based on receiver operating characteristic (ROC) curves for each TSM. All data here were derived from the training dataset. Bold: Marker of an isotope peak.

Supplementary Data 3. Tentative molecular assignment of taxon-specific markers. Isotope peaks, as identified from inspection of raw data, are indicated by bold font. Adduct ion formats were confirmed by isotopic pattern and can be unknown due to peak overlapping and low signal-to-noise ratio. Ions were identified using exact mass in the LIPID MAPS® structure database, Human Metabolome Database and Pseudomonas aeruginosa metabolite database. The last column shows the markers that were also detected by references. The references are listed at the end of the table. Only ID in mass tolerance of 10 ppm were considered. The identification confidence levels were decided as described by Schymanski et al.(Ref. 11). IDs at level 3 are verified by MS2. Orange: marker, its isotopic peak(s) was also detected as a TSM marker. Bold: Marker of an isotopic peak. Grey: tentative ID and molecular formula for markers of identification confidence level 5.

Supplementary Data 4. Metaspace and MetaboLights access links for imaging datasets. All samples used in the mass spectrometry imaging analysis are listed.

Supplementary Data 5. Comparison of number of taxon-specific markers detected by DESI-MSI and copy numbers of bacteria detected by 16S rRNA sequencing-based community analysis for the same colorectal tissue specimens on class-level.

Supplementary Data 6. Comparison of taxon-specific markers detected by DESI-MSI for the colorectal tissue specimens and those detected by LC-MS analysis in faecal samples.

Supplementary Data 7. Database composition of the external dataset of pure bacterial cultures recorded on a Xevo G2-XS instrument. Taxonomies from Gram to species are given.
