# Supplementary material for: Universal, untargeted detection of bacteria in tissues using metabolomics workflows
Source: Nat Commun. 2025 Jan 2;16:165. doi: 10.1038/s41467-024-55457-7 (PMC11697447; doi:10.1038/s41467-024-55457-7)
Supplement: Supplementary file 11 — Reporting Summary [file 41467_2024_55457_MOESM11_ESM.pdf]

nature portfolio

Corresponding author(s): Nicole Strittmatter
James McKenzie

Last updated by author(s): Nov 25, 2024

# Reporting Summary

Nature Portfolio wishes to improve the reproducibility of the work that we publish. This form provides structure for consistency and transparency in reporting. For further information on Nature Portfolio policies, see our Editorial Policies and the Editorial Policy Checklist.

## Statistics

For all statistical analyses, confirm that the following items are present in the figure legend, table legend, main text, or Methods section.

| n/a | Confirmed | |
|---|---|---|
| ☐ | ☒ | The exact sample size (*n*) for each experimental group/condition, given as a discrete number and unit of measurement |
| ☐ | ☒ | A statement on whether measurements were taken from distinct samples or whether the same sample was measured repeatedly |
| ☐ | ☒ | The statistical test(s) used AND whether they are one- or two-sided<br>*Only common tests should be described solely by name; describe more complex techniques in the Methods section.* |
| ☐ | ☒ | A description of all covariates tested |
| ☐ | ☒ | A description of any assumptions or corrections, such as tests of normality and adjustment for multiple comparisons |
| ☐ | ☒ | A full description of the statistical parameters including central tendency (e.g. means) or other basic estimates (e.g. regression coefficient) AND variation (e.g. standard deviation) or associated estimates of uncertainty (e.g. confidence intervals) |
| ☒ | ☐ | For null hypothesis testing, the test statistic (e.g. *F*, *t*, *r*) with confidence intervals, effect sizes, degrees of freedom and *P* value noted<br>*Give P values as exact values whenever suitable.* |
| ☒ | ☐ | For Bayesian analysis, information on the choice of priors and Markov chain Monte Carlo settings |
| ☐ | ☒ | For hierarchical and complex designs, identification of the appropriate level for tests and full reporting of outcomes |
| ☒ | ☐ | Estimates of effect sizes (e.g. Cohen's *d*, Pearson's *r*), indicating how they were calculated |

*Our web collection on statistics for biologists contains articles on many of the points above.*

## Software and code

Policy information about availability of computer code

| | |
|---|---|
| Data collection | Data collection was performed using vendor-specific instrument software. Raw data files were converted to MZ5 format using ProteoWizard msconvert version 3.0.4043. MS imaging files were converted to imzML format via imzML Converter (version 1.0.5). |
| Data analysis | All data analysis was performed using MATLAB R2023a and the Statistics and Machine Learning and Signal Processing toolboxes. The code used to determine the specificity of markers is available at https://github.com/jsmckenzie/bacterialTSM. |

For manuscripts utilizing custom algorithms or software that are central to the research but not yet described in published literature, software must be made available to editors and reviewers. We strongly encourage code deposition in a community repository (e.g. GitHub). See the Nature Portfolio guidelines for submitting code & software for further information.

## Data

Policy information about availability of data

All manuscripts must include a data availability statement. This statement should provide the following information, where applicable:

- Accession codes, unique identifiers, or web links for publicly available datasets
- A description of any restrictions on data availability
- For clinical datasets or third party data, please ensure that the statement adheres to our policy

The bacterial mass spectrometry data generated in this study have been deposited in the MetaboLights database under accession code MTBLS10328 [https://www.ebi.ac.uk/metabolights/MTBLS10328add hyperlink here]. The DESI mass spectrometry imaging data of human colorectal tissues data used in this study have been deposited in the MetaboLights database under accession code MTBLS289 [https://www.ebi.ac.uk/metabolights/editor/MTBLS289]. The DESI mass

spectrometry imaging data of murine tissues generated in this study have been deposited in the MetaboLights database under accession code MTBLS10846 [https://www.ebi.ac.uk/metabolights/MTBLS10846]. The mass spectrometry imaging data used in this study are also available in the METASPACE database [access link supplied in Supplementary Data 4]. The FISH data are available at Figshare [https://figshare.com/articles/figure/_/26504344]. The liquid chromatography-mass spectrometry (LC-MS) data for human faecal samples and the external bacterial mass spectrometry (REIMS) data used in this study are deposited (yet available undercurrently still in curation) under MetaboLights identifiers MTBLS11775XXXXX and MTBLS11776XXXXX, respectively.  restricted access to protect confidentiality and ensure appropriate data use. Access to these datasets can be requested by contacting the corresponding authors,Until publication, data can be requested from Prof. Dr. Nicole Strittmatter (nicole.strittmatter@tum.de) or Dr. James McKenzie (j.mckenzie@imperial.ac.uk). The 3274 bacterial isolates used to train and validate the TSM model are available at MetaboLights MTBLS10328. The DESI mass spectrometry imaging data of human colorectal tissues are available via MTBLS289. All DESI mass spectrometry imaging data from human and murine tissues can be accessed via MetaboLights MTBLS10846 and METASPACE (access link supplied in Supplementary Data 4). FISH results are uploaded in Figshare (https://figshare.com/articles/figure/_/26504344). Source data are provided with this paper.

## Research involving human participants, their data, or biological material

Policy information about studies with human participants or human data. See also policy information about sex, gender (identity/presentation), and sexual orientation and race, ethnicity and racism.

| | |
|---|---|
| Reporting on sex and gender | samples were taken from male and female participants. Info is included for LC-MS of fecal samples (see Supplmenetary Methods), gender was not recorded for CRC specimen used for DESI-MSI as it was deemed not relevant in relation to study outcomes |
| Reporting on race, ethnicity, or other socially relevant groupings | we did not collect information on ethnical background as it is not related to study objectives. |
| Population characteristics | we did not collect information on ethnical background as it is not related to study objectives. |
| Recruitment | Recruitment is detailed in Supplementary Methods on LC-MS and DESI-MSI |
| Ethics oversight | The use of human colorectal tissue specimen and faecal samples was ethically approved by the institutional review board at Imperial College Healthcare NHS Trust under reference numbers 07/H0712/112 and SUR_JK_17_046, respectively. |

Note that full information on the approval of the study protocol must also be provided in the manuscript.

# Field-specific reporting

Please select the one below that is the best fit for your research. If you are not sure, read the appropriate sections before making your selection.

[x] Life sciences   [ ] Behavioural & social sciences   [ ] Ecological, evolutionary & environmental sciences

For a reference copy of the document with all sections, see nature.com/documents/nr-reporting-summary-flat.pdf

# Life sciences study design

All studies must disclose on these points even when the disclosure is negative.

| | |
|---|---|
| Sample size | Bacteria were isolated during routine practice at the NWLP clinical microbiology laboratory at Charing Cross hospital, UK. This was a prospective study, so no sample size calculation was performed, and samples were analysed as and when available. This resulted in a total of 3274 independent bacterial strains spanning 232 species. Bacterial IDs were obtained using the Bruker Biotyper platform (identification through ribosomal protein profiles using MALDI-MS) by Biomedical Scientist microbiology staff during routine clinical microbiology practice following manufacturers protocols (expected misclassification rate 2-5%). |
| Data exclusions | There were no exclusions beyond currupted files (n=1) |
| Replication | Each isolate was analysed by 5 sampling events using the bipolar sampling probe. |
| Randomization | 597/3274 observations were partitioned into the training set, using each unique species and limiting the overall number of entries to a maximum of 7 per species. Different culturing media (rich media only), culturing ages and culturing atmospheres were included in the sample set where available. |
| Blinding | Blinding was not performed due to the objective nature of MS data acquisition. |

# Reporting for specific materials, systems and methods

We require information from authors about some types of materials, experimental systems and methods used in many studies. Here, indicate whether each material, system or method listed is relevant to your study. If you are not sure if a list item applies to your research, read the appropriate section before selecting a response.

## Materials & experimental systems

| n/a | Involved in the study |
| --- | --- |
| x | Antibodies |
| x | Eukaryotic cell lines |
| x | Palaeontology and archaeology |
| | x Animals and other organisms |
| | x Clinical data |
| x | Dual use research of concern |
| x | Plants |

## Methods

| n/a | Involved in the study |
| --- | --- |
| x | ChIP-seq |
| x | Flow cytometry |
| x | MRI-based neuroimaging |

# Animals and other research organisms

Policy information about studies involving animals; ARRIVE guidelines recommended for reporting animal research, and Sex and Gender in Research

| | |
| --- | --- |
| Laboratory animals | CRC: compound transgenic strain Apc1638N/wt x pvillin-KrasV12G on a C57/BL6N background; jejunin: BALB/c mouse; H. pylori: C57/BL6 |
| Wild animals | n/a |
| Reporting on sex | All miceused in this study were female (see Supplementary Methods) and Refs https://doi.org/10.1053/j.gastro.2006.08.011 and https://doi.org/10.1007/s00216-014-8237-2 |
| Field-collected samples | n/a |
| Ethics oversight | Bavarian Government (Regierung von Oberbayern) |

Note that full information on the approval of the study protocol must also be provided in the manuscript.

# Clinical data

Policy information about clinical studies

All manuscripts should comply with the ICMJEguidelines for publication of clinical research and a completedCONSORT checklist must be included with all submissions.

| | |
| --- | --- |
| Clinical trial registration | no data from clinical trials was used in this study. |
| Study protocol | n/a |
| Data collection | n/a |
| Outcomes | n/a |

# Plants

| | |
| --- | --- |
| Seed stocks | n/a |
| Novel plant genotypes | n/a |
| Authentication | n/a |
